# Bioinspired Materials 2018: Conference Report

**DOI:** 10.3390/biomimetics4010004

**Published:** 2019-01-14

**Authors:** Marloes Peeters, Patricia Linton, Araida Hidalgo-Bastida

**Affiliations:** 1 Division of Chemistry and Environmental Science, Faculty of Science and Engineering, Manchester Metropolitan University, Chester Street, Manchester M15GD, UK; 2 Division of Biology, Faculty of Science and Engineering, Manchester Metropolitan University, Chester Street, Manchester M15GD, UK; p.e.linton@mmu.ac.uk; 3 Division of Biomedical Sciences, School of Healthcare Sciences, Manchester Metropolitan University, Chester Street, Manchester M15GD, UK; a.hidalgo@mmu.ac.uk

**Keywords:** biomimetics, bioinspired materials, tissue engineering, hydrogels, sensors, materials chemistry, nanomaterials, bioengineering

## Abstract

The Bioinspired Materials conference 2018 was organized for the third time by a team of researchers from Manchester Metropolitan University. This international conference aims to bring together the scientific committee in the fields of biomimetic sensors, bioinspired materials, materials chemistry, three-dimensional (3D) printing, and tissue engineering. The 2018 edition was held at the John Dalton Building of Manchester Metropolitan University, Manchester, UK, and took place on the 10th of October 2018. There were over 60 national and international attendees, with the international attendees participating in a lab tour through the synthetic facilities and Fuel Cell Innovation Centre on the 9th of October. The three conference sessions encompassed a wide range of topics, varying from biomimetic sensors, hydrogels, and biofabrics and bioengineering.

## 1. Introduction

In 2016, the first Bioinspired Materials conference was organized by researchers (Dr. Patricia Linton, Dr. David Sawtell, Dr. Mikhajlo Zubko, Dr. Araida Hidalgo-Bastida, and Dr. Marloes Peeters) from Manchester Metropolitan University from within the schools of Science & Engineering and Healthcare Science. The event featured three invited speakers and a poster session, which was attended by fifty local attendees. Due to the great success of the first symposium, the 2017 event was extended to a two-day conference and sponsored by the Royal Society of Chemistry’s Materials Division. This conference, with over 60 international attendees, had internationally recognized keynote speakers and twenty oral presentations. On the first day, there was a dinner and networking event which allowed participants to forge international and multidisciplinary collaborations. Due to the positive feedback received from international visitors, it was decided that the 2018 event would be hosted again at Manchester Metropolitan University. This year’s conference was over the course of two days, with one day of lab tours and a social event for international visitors (including from Japan and Bangladesh) and one day of talks and posters. Therefore, the conference was a perfect mix of local and international visitors with expertise in various areas, ranging from engineering to healthcare, microbiology, chemistry, and material science.



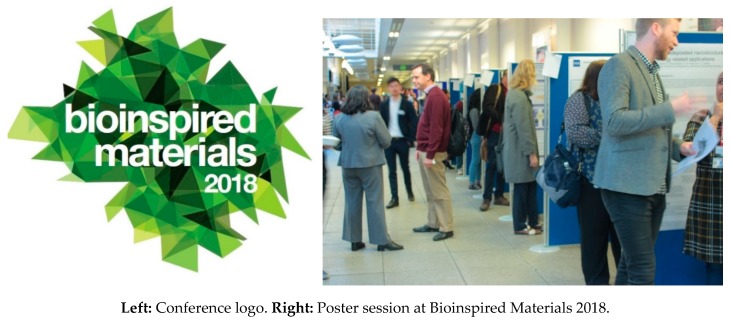

**Left**: Conference logo. **Right**: Poster session at Bioinspired Materials 2018.

## 2. Sessions

In this conference report, we will summarize the key content and topics presented at the conference, with 15 oral contributions (three plenary lectures) and 27 poster presentations in total. 

### 2.1. Session 1: Biomimetic Sensors and Scaffolds

The conference was opened by Prof. Patrick Wagner from the Laboratory of Soft Matter and Biophysics at KU Leuven. His research focuses on the use of synthetic receptors, such as molecularly imprinted polymers (MIPs) and surface imprinted polymers (SIPs), for the detection of biomolecules [1]. The use of these biomimetic receptors has the advantages of affordability and superior thermal and chemical stability compared to antibodies. He discussed a range of measurement techniques that can be used in combination with these recognition elements, such as the heat-transfer method (HTM), gravimetric methods, and electrochemical impedance spectroscopy. This concept of MIPs came back in other presentations during this session, including that of Dr. Pijush Kumar [2] discussing their use in drug delivery, and Joseph Lowdon reporting on the use of new MIP functionalization strategies [3]. 



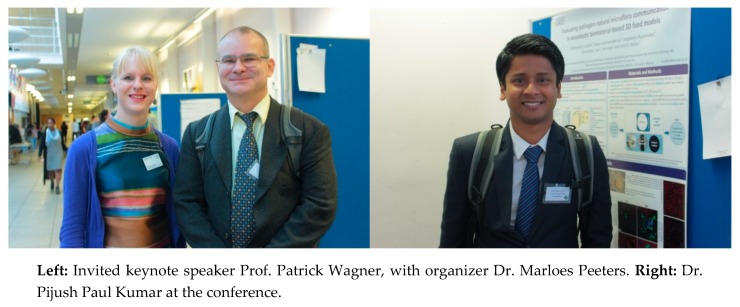

**Left**: Invited keynote speaker Prof. Patrick Wagner, with organizer Dr. Marloes Peeters. **Right**: Dr. Pijush Paul Kumar at the conference.

Prof. Mun’de Vestergaard discussed a different bio-inspired technology, the use of membranes, to develop biosensors [4]. Biomimetics scaffolds composed of whey proteins were discussed by Dr. Karl Norris [5] and Anna-Maria Tryba [6], while Dr. Priyanka Gupta [7] focused on the use of polyurethane to create three-dimensional (3D) tumour model systems. 

### 2.2. Session 2: Hydrogels

The session on hydrogels was opened by an invited plenary lecture of Prof. Alberto Saiani from the University of Manchester [8]. His research focuses on peptide hydrogel technology, which he has successfully commercialized. The use of polyurethane hydrogels and scaffolds was presented by Stella Totti [9]. Benjamin Filby [10] talked about the use of Pickering emulsion to form hydrogels, while Dr. Alison Edwards [11] discussed the design of monosaccharide amphiphiles as hydrogelators. 

### 2.3. Session 3: Biofabrics and Bioengineering

The final session of the conference focused on various aspects of bioengineering. Jane Wood [12] from Manchester Metropolitan University was invited to present her research on bioinspired clothing. She was wearing one of her bioinspired garments that was developed using bacteria. This was followed by Prof. Vesselin Paunov [13], who demonstrated that the antimicrobial effect of berberine and chlorhexidine can be enhanced by using nanogel carriers. Dr. Gethin Allen [14] showcased synthetic biology-inspired, protein-based compounds that can be implemented as corrosion-resistant coatings. The use of bioinspired technology in sports was discussed by Dr. Tom Allen [15], who brought along 3D-printed structures that are used in sports clothing and equipment. The final talk of the day was by Prof. Dou Zhang from the Institute of Powder Metallurgy based in Central South University in China. 

## 3. Poster Contributions

There were over 27 presentations covering a range of topics. The 1st prize, sponsored by Fluorochem, was won by Dr. Sachin N. Shah [16] from the University of Hull for his work on the synthesis of nanocomposite materials from plant viruses. The runner-up prize was for Cosimo Ligorio from the University of Manchester [17]. The best poster presentation prize for participants from Manchester Metropolitan University was awarded to Dr. Alex Hudson [18], who presented the development of polymer-based sensor platforms for the thermal detection of proteins.

## 4. Conclusions

Bioinspired Materials 2018 was a great success, and through this conference we were able to bring together a group of diverse multidisciplinary scientists and forge new collaborations. We look forward to continuing with the event and hosting it again at Manchester Metropolitan University in 2019.
